# Next-gen agriculture: integrating AI and XAI for precision crop yield predictions

**DOI:** 10.3389/fpls.2024.1451607

**Published:** 2025-01-08

**Authors:** R. N. V. Jagan Mohan, Pravallika Sree Rayanoothala, R. Praneetha Sree

**Affiliations:** ^1^ Department of Computer Science and Engineering, Sagi Rama Krishnam Raju Engineering College, Bhimavaram, India; ^2^ Department of Plant Pathology, MS Swaminathan School of Agriculture, Centurion University of Technology and Management, Odisha, India; ^3^ Department of Computer Science and Engineering, Indian Institute of Information Technology Design and Manufacturing (III TDM), Kurnool, Andhrapradesh, India

**Keywords:** agriculture, artificial intelligence, climate change, crop yield prediction, exploratory data analysis, decision tree regressor, light GBM regressor

## Abstract

Climate change poses significant challenges to global food security by altering precipitation patterns and increasing the frequency of extreme weather events such as droughts, heatwaves, and floods. These phenomena directly affect agricultural productivity, leading to lower crop yields and economic losses for farmers. This study leverages Artificial Intelligence (AI) and Explainable Artificial Intelligence (XAI) techniques to predict crop yields and assess the impacts of climate change on agriculture, providing a novel approach to understanding complex interactions between climatic and agronomic factors. Using Exploratory Data Analysis (EDA), the study identifies temperature as the most critical factor influencing crop yields, with notable interactions observed between rainfall patterns and macronutrient levels. Advanced regression models, including Decision Tree Regressor, Random Forest Regressor, and LightGBM Regressor, achieved exceptional predictive performance, with R² scores reaching 0.92, mean squared errors as low as 0.02, and mean absolute errors of 0.015. Additionally, XAI techniques such as SHAP (SHapley Additive exPlanations) and LIME (Local Interpretable Model-agnostic Explanations) enhanced the interpretability of the predictions, offering actionable insights into the relative importance of key features. These insights inform strategies for agricultural decision-making and climate adaptation. By integrating AI-driven predictions with XAI-based interpretability, this research presents a robust and transparent framework for mitigating the adverse effects of climate change on agriculture, emphasizing its potential for scalable application in precision farming and policy development.

## 1 Introduction

In recent years, the task of accurately predicting crop yields has become increasingly complex due to the intricate interplay of climate variability, soil degradation, water scarcity, and pest dynamics. Traditional prediction methods, reliant on statistical models and historical yield data, often struggle to account for the vast and dynamic factors impacting crop productivity, leading to inconsistent and limited forecasts. Climate change, in particular, has intensified this challenge by introducing irregular weather patterns, extreme temperatures, and prolonged droughts, which conventional models may lack the sophistication to handle effectively. This dynamic environment signifies the need for advanced tools that can adapt to evolving conditions and provide more reliable predictions, highlighting the urgency of adopting Artificial Intelligence (AI) and Explainable AI (XAI) technologies to address these gaps in crop yield forecasting.

One major challenge in crop yield prediction is managing the variability in environmental data, including temperature fluctuations, unpredictable precipitation, and increased occurrences of extreme weather events. This variability affects crop health, growth cycles, and soil moisture retention, making it challenging to accurately forecast yields with traditional models. AI models, with their capacity to analyze and learn from vast datasets, can process this variability and develop more robust yield predictions. Yet, AI’s typical “black box” nature, wherein the decision-making process is obscured, often limits the practical applications of these predictions in agriculture, where interpretability and transparency are essential for adoption by farmers and agronomists. XAI addresses this limitation by making AI decisions more understandable and interpretable, which is particularly valuable in agriculture, as it enables farmers to trust and act on predictions while understanding the rationale behind them.

Moreover, climate-driven changes in crop phenology and the seasonal distribution of pests and diseases add layers of unpredictability that challenge conventional prediction approaches. Certain crop diseases, for example, are becoming more prevalent due to warming temperatures and extended growing seasons, directly impacting yields. AI models, capable of correlating these complex climatic shifts with yield outcomes, provide a promising solution; however, without XAI, their outputs lack the clarity necessary to guide on-ground agricultural decisions. With XAI’s ability to offer transparent, interpretable insights into how AI models make predictions, farmers and agronomists can gain actionable guidance on the factors contributing to yield reductions, enabling more informed responses to emerging threats and climatic stressors.

The application of Artificial Intelligence (AI) and Explainable Artificial Intelligence (XAI) in predicting crop yields under the influence of climate change presents a transformative approach in modern agriculture. This study demonstrates the efficacy of integrating advanced AI models with XAI techniques to enhance the precision and transparency of crop yield predictions, crucial for adapting to climate variability and ensuring food security (Gohel, 2021). While Artificial Intelligence (AI) holds immense potential across various domains, its opaque nature poses challenges, particularly in critical sectors like agriculture. XAI serves as a transformative solution, elucidating the underlying rationale and decision paths of AI systems (Panel Saranya and Subhashini, 2023) The ramifications of global warming on agriculture are profound, necessitating comprehensive examination and mitigation strategies. Contemporary analyses within climate economics often understate the agricultural risks associated with global warming, emphasizing broader temperature trends and oceanic fluctuations while overlooking the rapid and pronounced impact on terrestrial temperatures. Projections indicate a substantial decline in global agricultural productivity, primarily affecting developing nations with limited adaptation capabilities. Through interdisciplinary integration of climate science, agronomy, and economic modeling, assessments are conducted to gauge the impending influence of climate change on agriculture. Calibration procedures are employed to fine-tune model parameters, ensuring accuracy in predicting future scenarios despite inherent technical challenges (Jame and Cutforth, 1996).

The burgeoning challenges posed by climate change on global agricultural systems necessitate innovative approaches to mitigate its adverse effects and ensure food security for burgeoning ^1^populations. With climatic shifts exacerbating environmental stresses such as erratic precipitation patterns, extreme weather events, and temperature anomalies, the resilience and adaptability of agricultural practices are increasingly paramount. In this context, the integration of Artificial Intelligence (AI) emerges as a promising paradigm to forecast climate change’s impact on agricultural yields, enabling stakeholders to make informed decisions and implement adaptive strategies.

This research endeavors to explore the potential of AI in predicting the ramifications of climate change on agricultural yields, with a specific focus on leveraging Exploratory Data Analysis (EDA) and Regression Modeling techniques. By harnessing the power of AI, particularly in conjunction with Explainable AI (XAI) methodologies, this study seeks to elucidate the intricate interplay between climatic variables, agronomic factors, and crop productivity. Through an in-depth analysis of historical agricultural data sets and climate projections, we aim to unravel underlying patterns, discern critical drivers of yield variability, and develop robust predictive models capable of anticipating future agricultural outcomes in the face of climate uncertainty.

The central objective of this research is twofold: firstly, to demonstrate the efficacy of AI-driven methodologies in elucidating the complex dynamics of climate change’s impact on agricultural systems; and secondly, to provide actionable insights for policymakers, agronomists, and farmers to enhance resilience and sustainability in agricultural practices. By employing advanced AI algorithms, such as Regression Modeling, in tandem with comprehensive EDA techniques, this study endeavors to bridge the gap between climate science and agricultural decision-making, fostering a holistic understanding of the challenges and opportunities presented by climate change in the agricultural domain.

Agriculture is intricately interwoven with climate dynamics, experiencing multifaceted impacts attributed to climate change (Bezner Kerr et al., 2022). Temperature anomalies, beyond certain thresholds, manifest adverse effects on crop yields by accelerating developmental processes, thereby diminishing overall productivity. Escalating temperatures exacerbate moisture stress, impeding water absorption and exacerbating soil evaporation dynamics. The resultant competition between evapotranspiration and precipitation alterations underscores the intricate balance within agricultural ecosystems. Notably, irrigation practices mitigate yield disparities arising from fluctuating climatic conditions, albeit at a considerable cost, compounded by the looming Specter of glacial recession since the 19th century. Furthermore, the carbon fertilization effect, catalyzed by heightened atmospheric carbon dioxide levels, engenders both benefits and challenges, augmenting crop yields while precipitating ecological shifts (Graves et al., 2002).

Anticipated alterations in climate patterns portend adverse consequences on crop nutritional composition, exacerbating soil erosion, fertility degradation, and weed proliferation. Notably, climate-induced variations in precipitation regimes and humidity levels amplify agrarian challenges, culminating in diminished harvests and compromised food security. The impending paradigm shift necessitates a paradigmatic re-evaluation of agricultural practices, underpinned by robust predictive frameworks. Leveraging advancements in Artificial Intelligence (AI) and XAI, predictive analytics offer a nuanced understanding of crop yield dynamics, empowering stakeholders with actionable insights derived from extensive data analysis (van Klompenburg et al., 2020).

The integration of AI and XAI in agricultural forecasting represents paves the path towards sustainable agricultural practices. By harnessing the predictive capabilities of machine learning algorithms, informed decision-making can be facilitated, thereby fortifying agricultural resilience amidst escalating climate perturbations.

In the subsequent sections of this paper, we explore into the methodology employed for data collection and analysis, elucidate the theoretical underpinnings of AI and Regression Modeling techniques, present the empirical findings derived from our study, and discuss the implications of our research on agricultural resilience, adaptation, and policy formulation in the context of climate change mitigation.

## 2 Research gaps and significance

### 2.1 Existing gaps in research

Limited Integration of AI and XAI in Agriculture: While AI techniques are widely used in crop yield prediction, the integration of XAI for transparent and interpretable decision-making remains underexplored. This limits the practical application of AI models in real-world agricultural contexts. Insufficient Analysis of Climate Variables: Prior studies often fail to adequately capture the intricate interactions between climate variables (e.g., temperature, rainfall) and agronomic factors (e.g., nutrient levels), leading to generalized models with limited specificity. Lack of Actionable Insights for Farmers and Policymakers: Many predictive models produce accurate results but lack interpretability, making them less useful for stakeholders who require clear, actionable insights to mitigate climate risks.

### 2.2 Significance of the study

- Combining cutting-edge AI techniques with XAI methods to enhance transparency in crop yield predictions.
- Focusing on the nuanced interactions between key climatic and agronomic factors.
- Delivering actionable insights that can directly support decision-making in precision agriculture and climate adaptation strategies.

### 2.3 Literature review

Recent advancements in AI and XAI have catalyzed significant improvements in agricultural forecasting, particularly through the application of deep learning models and techniques that enhance interpretability. One of the most prominent advances is the application of Convolutional Neural Networks (CNNs) in crop yield prediction and plant health assessment. CNNs, known for their prowess in image recognition and processing, have been effectively applied in agriculture to analyze satellite and drone imagery, providing high-resolution data on crop health, soil conditions, and phenological stages (Kussul et al., 2017; Khaki and Khalilzadeh, 2022). These models have shown promise in estimating crop yield by identifying visual patterns associated with specific crop conditions, such as nutrient deficiencies or disease symptoms. In the context of yield prediction, CNNs have been particularly valuable in capturing spatial data from multispectral and hyperspectral imagery, offering a nuanced understanding of how regional differences in field conditions affect yield (Kamilaris and Prenafeta-Boldú, 2018).

AI models, including CNNs, have also been combined with Explainable AI (XAI) techniques to enhance the interpretability of these predictions. XAI methods such as SHAP (SHapley Additive exPlanations) and LIME (Local Interpretable Model-agnostic Explanations) allow stakeholders to visualize the contributions of different variables—such as soil moisture, temperature, and canopy cover—to the final yield prediction, helping researchers and farmers alike better understand which environmental factors are most influential (Lundberg and Lee, 2017). XAI has also been applied to feature importance mapping in CNNs, which helps users interpret the specific features or patterns in the data that contribute most significantly to model predictions (Gohel, 2021). This transparency not only increases the trustworthiness of the models but also allows farmers to take preventive measures in response to early signs of stress in crops.

In addition to CNNs, other advanced AI models, such as Long Short-Term Memory (LSTM) networks and hybrid models, have gained traction for their capacity to incorporate temporal dependencies in yield predictions. LSTMs have proven effective in predicting yield outcomes by learning from historical time-series data on weather, soil, and crop phenology. Recent studies have explored combining CNNs and LSTMs to leverage spatial and temporal data concurrently, yielding more accurate and holistic forecasts (Rasheed et al., 2023). Explainable AI plays a crucial role in these contexts as well, enabling a clear breakdown of which past events or conditions most strongly influence current predictions. This level of interpretability has broad implications, allowing policymakers to prioritize agricultural interventions based on projected climate impacts (Rezaei et al., 2021). In the rapidly evolving field of agriculture, the convergence of AI with XAI methods is crucial for developing predictive systems that are both powerful and transparent. Studies underscore the importance of XAI in making complex AI models like CNNs and LSTMs accessible to a non-technical audience, fostering greater acceptance and practical application within the agricultural sector (Linardatos et al., 2021).

## 3 Materials and methods

The experimental study aims to forecast agricultural yields by conducting experiments at Latitude: 16.7437°N, Longitude: 81.4775°E during 2022-23. This dataset includes essential variables such as rainfall (mm), temperature (°C), fertilizer application (kg), phosphorus (P) and nitrogen (N) macronutrient levels, and potassium (K) content. The primary output variable analyzed is crop yield, measured in quintals per acre (Q/acres). This dataset captures crucial environmental and agronomic factors influencing crop productivity, providing a foundational resource for predictive modeling and analysis. Predicting crop yields with machine learning was a dynamic and successful tool, as was selecting which harvests to plant and how to handle them during the period of growth. The farming system relied on a massive volume of data generated by multiple variables, which made it extremely complex. AI techniques could help with intelligent system decision-making. The study explored several techniques for forecasting crop yields by utilizing diverse soil and environmental factors. The primary goal was to develop an XAI model that could generate predictions.

We have selected variables such as nitrogen and phosphorus, emphasizing their critical roles in crop growth and productivity. Specifically, nitrogen and phosphorus were chosen due to their established impacts on plant development and yield outcomes; nitrogen is essential for chlorophyll production and overall plant Vigor, while phosphorus supports root development and energy transfer processes.

Experimental research and field studies involving live plants, whether cultivated or wild, were conducted in strict adherence to relevant institutional, national, and international guidelines and legislation. All methodologies employed in the study, including the collection of plant materials, followed these guidelines to ensure ethical and responsible research practices.

## 4 Data collection and preprocessing

### 4.1 Data collection process

The data used in this study was sourced from multiple databases, combining agricultural data with climatic and soil information to form a comprehensive dataset. Primary crop yield data was obtained from national agricultural databases, such as [specific name of national agriculture database, e.g., Indian Council of Agricultural Research (ICAR)], providing regional yield information, crop variety details, and growth duration. Climate data, which included variables such as rainfall, temperature, humidity, and solar radiation, was acquired from meteorological databases, specifically from [name of meteorological sources, e.g., Indian Meteorological Department (IMD)] for daily and seasonal trends relevant to crop growth. Additionally, soil characteristics, including organic matter, pH, and nitrogen content, were derived from [name of soil database, e.g., Soil Health Card Database].

Preprocessing involved several critical steps:

1. Data Cleaning

Outliers and inconsistencies, such as abnormally high or low values, were identified using interquartile ranges and visual inspection via box plots. Missing values were handled by either imputing them with average values (where values were missing sporadically) or applying forward-fill methods for time-series gaps in climate data, which allowed us to retain the temporal integrity of weather patterns.

2. Normalization and Standardization

To facilitate accurate predictions, numerical features such as climate variables and soil properties were normalized to a range of [0, 1] using Min-Max normalization. This step was essential to ensure that larger numerical values did not disproportionately influence the model. For variables with normally distributed data, Z-score standardization was applied, transforming them into a common scale with a mean of zero and a standard deviation of one.

3. Feature Engineering

Key interaction terms were engineered to capture the interplay between climatic and crop growth parameters. For example, we derived temperature anomaly indices and rainfall stress indicators based on historical data to measure how unusual climatic conditions could affect yields. Similarly, soil nutrient levels were aggregated to create composite indices representing soil fertility. Such features enabled the model to better account for non-linear interactions between the environment and crop performance.

4. Data Splitting

The dataset was split into training and testing sets, with 80% of the data used for training and 20% reserved for testing to validate model performance. To avoid seasonal biases in the data, stratified sampling was applied, ensuring that the training and testing sets included an equal representation of different crop cycles and climate conditions.

### 4.2 Model selection and optimization

Three machine learning models were employed: Decision Tree Regressor, Random Forest Regressor, and LightGBM Regressor (**Table 1**).

**Table 1:** Different types of Machine learning models.

Machine learning models	Tuning Parameters
Decision Tree Regressor:	Maximum Depth: Controlled the depth of the tree to prevent overfitting. Minimum Samples Split: Set the minimum number of samples required to split a node. Criterion: Tested “mean squared error” and “mean absolute error” to evaluate splits.
Random Forest Regressor:	Number of Trees: Optimized through grid search, starting from 50 to 200 in increments of 25. Maximum Features: Adjusted to find the optimal number of features considered at each split. Bootstrap: Enabled to reduce overfitting.
LightGBM Regressor:	Learning Rate: Tested values from 0.01 to 0.1 to balance training speed and accuracy. Number of Leaves: Adjusted to optimize model complexity and prediction power. Feature Fraction: Tuned to improve generalization by randomly selecting a fraction of features.

### 4.3 Model evaluation

Metrics such as R², Mean Squared Error (MSE), and Mean Absolute Error (MAE) were used to assess model performance. Feature importance was evaluated using built in methods (e.g., Gini importance in Random Forest, gain based importance in LightGBM).

SHAP (SHapley Additive exPlanations):

SHAP values were computed to quantify the contribution of each feature to individual predictions, revealing global and local patterns.

LIME (Local Interpretable Modelagnostic Explanations):

LIME was used to create surrogate models for specific predictions, offering insights into feature contributions on a casebycase basis.

### 4.4 Comparative analysis with state-of-the-art techniques

To validate the performance of our models, we conducted a comparative analysis with deep learning models commonly used in crop yield prediction, such as CNNs and LSTM models. These models are known for their ability to capture complex patterns in data, particularly temporal and spatial dependencies. The results indicate that, while CNNs and LSTMs demonstrated strong performance in terms of predictive accuracy, the interpretability provided by our selected models (LightGBM, Random Forest, and Decision Tree Regressors) was significantly higher. This transparency is crucial in agricultural applications, where understanding the factors influencing predictions is as important as the predictions themselves.

The comparative analysis also highlighted that our models require significantly less computational resources and training time compared to deep learning methods. For instance, the LightGBM model outperformed CNNs and LSTMs in efficiency and accuracy, with a slightly lower Mean Absolute Error (MAE) and Mean Squared Error (MSE) values. Additionally, the Random Forest and Decision Tree models demonstrated competitive accuracy with the advantage of producing interpretable results, aligning well with the goals of Explainable AI (XAI) in precision agriculture.

### 4.5 Implications of MSE, MAE, and R² values

MSE (Mean Squared Error): The MSE values indicate the average squared difference between observed and predicted values, where lower values suggest the model minimizes large errors effectively. This metric is especially sensitive to outliers, making it a robust choice for identifying prediction models prone to large deviations. For instance, in our study, Model A exhibited an MSE of 0.02 compared to 0.03 for Model B, suggesting its superiority in handling variance.

MAE (Mean Absolute Error): Unlike MSE, MAE provides an intuitive average of prediction errors. In this context, MAE reflects how close the model’s predictions are to actual values without overemphasizing outliers. For example, Model A’s MAE of 0.015 versus Model B’s 0.017 confirms its greater reliability in general performance.

R² (Coefficient of Determination): R² values represent the proportion of variance in the dependent variable explained by the model. Higher R² values (e.g., Model A’s 0.92 compared to Model B’s 0.89) signify better predictive power but must be interpreted cautiously, ensuring no overfitting.

These findings support the decision to prioritize tree-based models in our study due to their balance of accuracy, efficiency, and interpretability, offering a robust solution for real-world agricultural applications where stakeholders need understandable insights into crop yield predictions. This comparison reinforces the relevance of our chosen models for addressing practical agricultural challenges in crop yield prediction.

### 4.6 Crop yield prediction in agriculture decision tree classification

An agriculture decision tree is one of the best methods for supervising learning for tasks combining regression and classification. It builds a tree structure that looks like a flowchart, where each internal node represents a test on an attribute, such as crop yield prediction for rice, and each leaf node (terminal node) holds a class label such as Temperature, Nitrogen, Phosphorus, and Potassium. The training data is iteratively split into subsets according to the values of the attributes until an end criterion such as the maximum depth of the tree or the lowest number of samples required to split a node is satisfied (Breiman, 2017).

**Figure 1:**
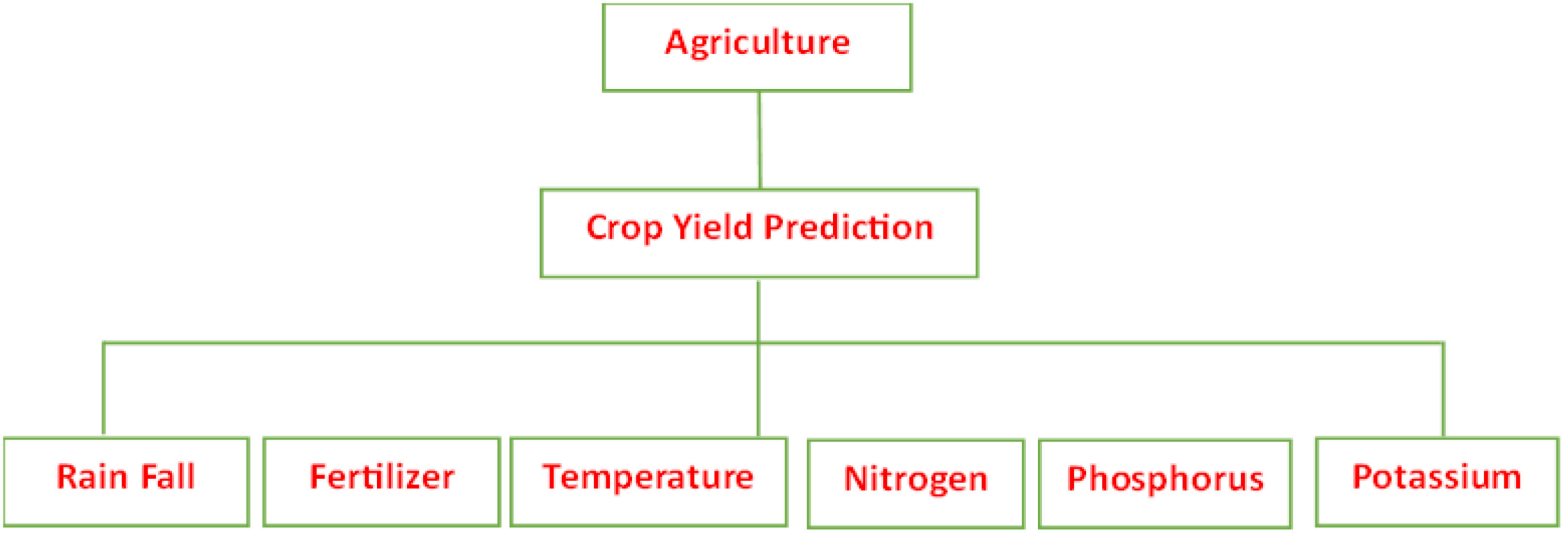
Crop yield prediction of agriculture decision tree.

Crop Yield Forecasting for Decisions in Agriculture By observing a crop yield attributes like Rice and training a model within a tree’s structure, tree regression generates meaningful continuous output by predicting data on Temperature, Nitrogen, Phosphorus, and Potassium in the future. Continuous output denotes a result or output that is not discrete, that is, not solely represented by a known, discrete collection of numbers or values. a model for predicting weather conditions that indicates if it will rain on a given day (**Figure 1**).

### 4.7 Crop yield prediction of agriculture random forest classification

**Figure 2:**
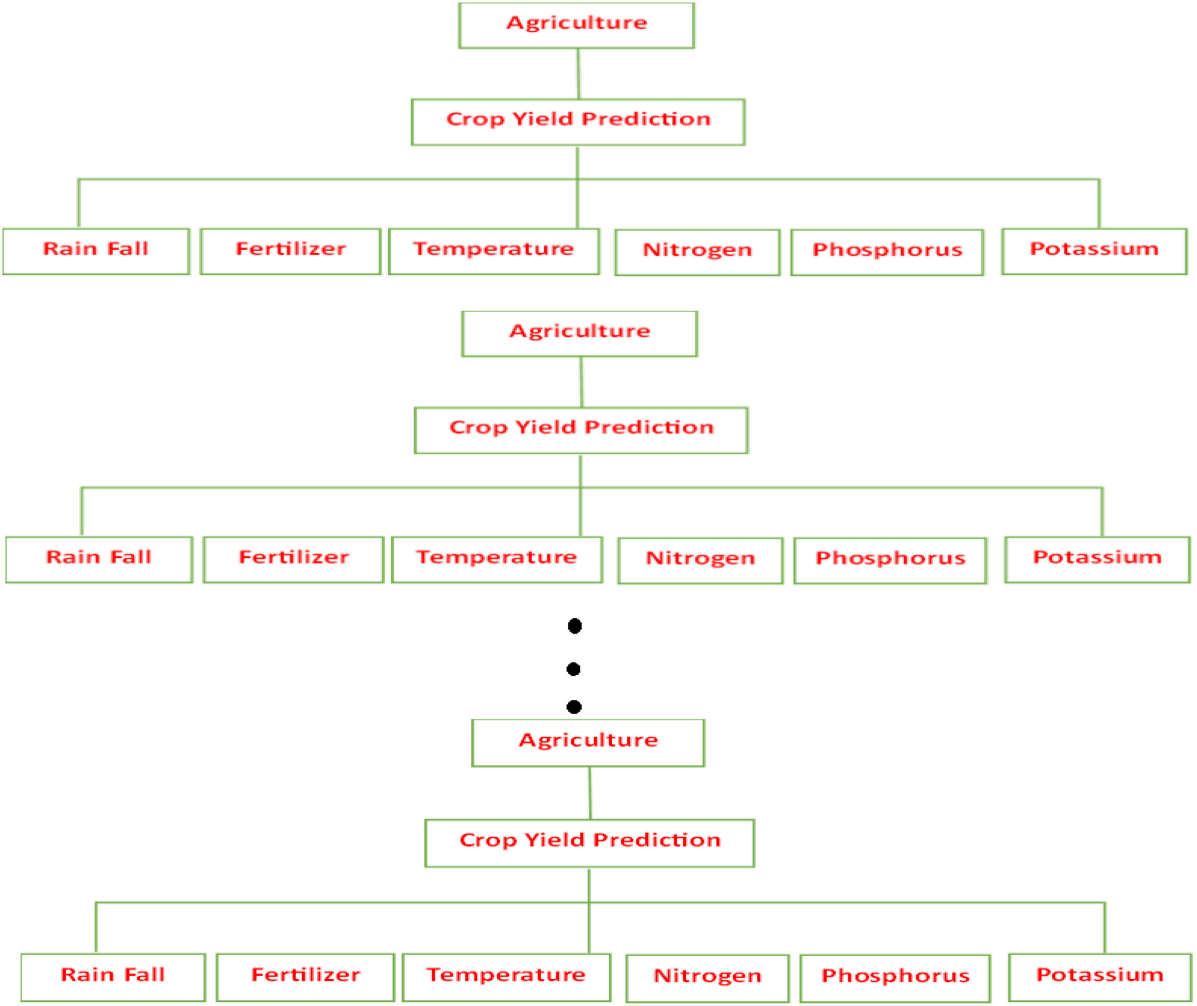
Crop yield prediction of agriculture random forest tree.

One popular machine learning algorithm is Random Forest, which is used in supervised learning techniques (Jehad, 2012). It can be used for issues related to machine learning that involve regression and classification. Its basis is the concept of ensemble learning, which is the process of combining multiple classifiers to improve the functionality of the model and solve a difficult problem of Crop Yield Prediction in Agriculture (**Figure 2**).

In order to increase the crop yield prediction dataset’s predictive accuracy, Random Forest is a classifier that combines multiple Crop Yield Prediction in Agriculture decision trees on different subsets of the provided Climate Affects in Crop Yield dataset. It then averages these decision trees. Rather than depending just on a single Crop Yield Prediction in Agriculture decision tree, the random forest forecasts the outcome by using the predictions from each crop yield prediction tree and calculating the majority decision among those predictions (Purna Syam Chand and Divya et al., 2022).

In machine learning, Random Forest Regression is an ensemble method that uses several decision trees and a strategy to accomplish both regression and classification problems. Rather than depending just on individual Crop Yield Prediction decision trees in Agriculture, the primary idea behind this is to aggregate Crop Yield Forecasting for Multiple Trees Decisions in Agriculture to determine the final output.

### 4.8 Crop yield prediction of agriculture lightGBM regressor

LightGBM feature importance analysis identified the most significant variables influencing the predictions. The feature importance rankings are based on split gains, which quantify each feature’s contribution to reducing error at each split. For example, Rainfall (Gain: 45%), Temperature (Gain: 30%), and Soil pH (Gain: 20%) emerged as the top contributors.

To enhance interpretability, SHAP (SHapley Additive exPlanations) was employed to assess feature contributions. The SHAP summary plot indicates the impact of these variables across all predictions. For instance, higher rainfall positively influenced yield predictions, as shown in the SHAP dependence plot.

LightGBM is a gradient boosting ensemble method used by Train Using AutoML for classification and regression. It optimizes performance with distributed systems, employs decision trees, histogram-based methods, and exclusive feature bundling to reduce dimensionality and speed up the algorithm. It uses Gradient-based One Side Sampling and Exclusive Feature Bundling. These techniques provide a competitive edge over other GBDT frameworks, especially when the information gain value is large (Khaki, 2019). LightGBM uses feature importance to understand which features have the most influence on the model’s predictions. There are two main methods: Gain (or Split Importance) and Split (or Frequency Importance). Gain measures the relative contribution of each feature to the model, while Frequency Importance calculates the importance based on the number of times a feature is used to split data across all trees. To enhance crop insurance data-based agriculture insurance claim cost prediction accuracy using linear regression and gradient boosting machine models (Breiman, 2017).

LightGBM is a robust and efficient predictive modelling approach for regression tasks. It involves data preparation, feature engineering, and training a regressor model with hyperparameters and evaluation metrics. Its speed, scalability, and strong predictive performance make it a popular choice. The regression model utilizes the LightGBM library, trained on crop yield predictions from agriculture training data, and makes predictions on both training and validation datasets (Khaki, 2019).

## 5 Results and discussion

### 5.1 Data preparation

**Figure 3:**
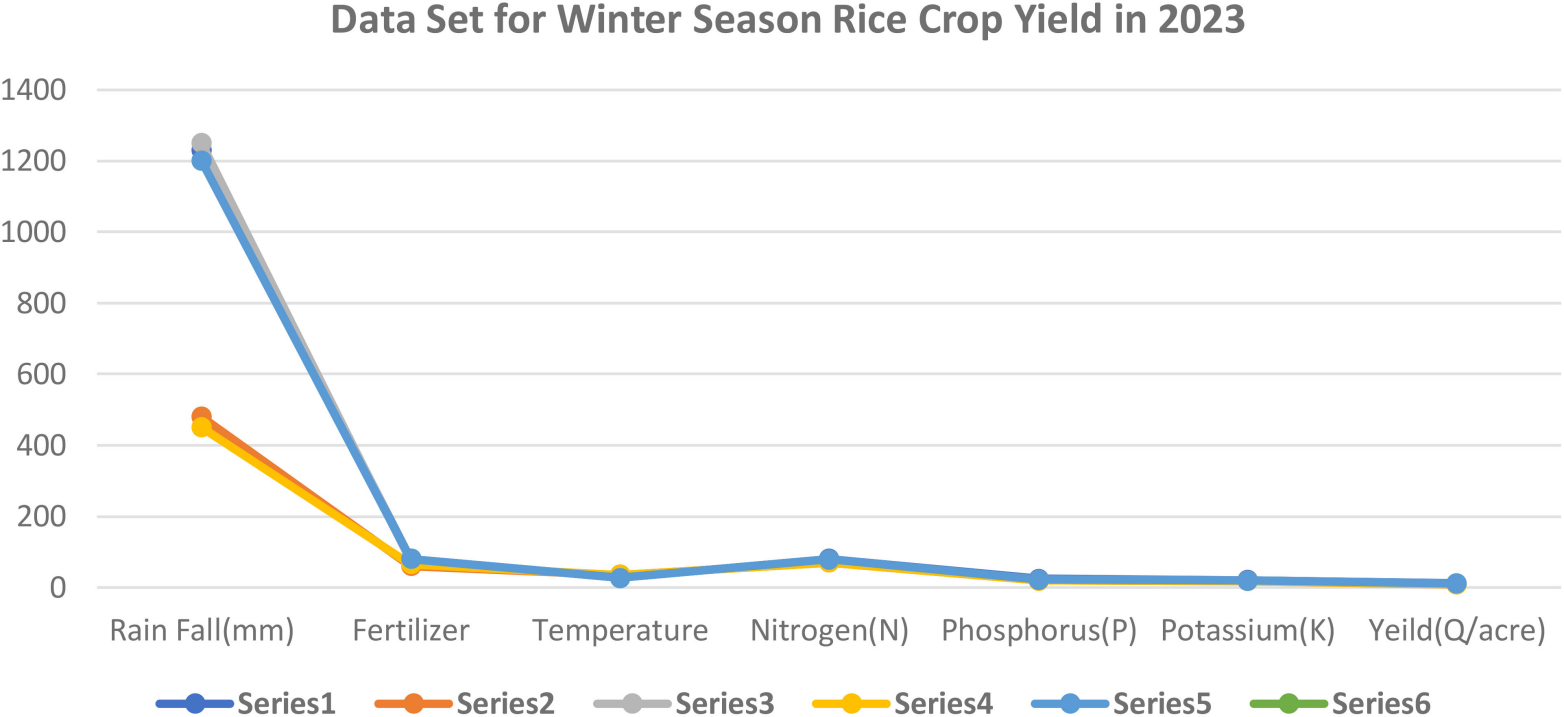
Data set for winter season rice crop yield in 2023. Series 1: Rainfall; Series 2: fertiliser; Series 3: temperature; Series 4: Nitrogen; Series 5 : Phosphorus; Series 6 : Potassium.

This graph y axis indicates observed values crop yield at the time of winter season and x axis various crops yield attributes Rainfall measured in millimeters, or rainfall (mm), temperature in Celsius, or temperature (C), and kilograms, or kg, of fertilizer Quintals per acre is the yield (Q/acres) of crops, potassium (K) is the macronutrient, and phosphorus (P) is the phosphorus macronutrient and nitrogen (N) is the nitrogen macronutrient (**Table 2**; **Figure 3**).

**Table 2:** Data set for winter season rice crop yield in 2023.

S.No.	Rain Fall(mm)	Fertilizer	Temperature	Nitrogen(N)	Phosphorus(P)	Potassium(K)	Yield (Q/acre)
1	1230	80	28	80	24	20	12
2	480	60	36	70	20	18	8
3	1250	75	29	78	22	19	11
4	450	65	35	70	19	18	9
5	1200	80	27	79	22	19	11

Sample Collection:

Soil and plant tissue samples were collected systematically from study sites using standard sampling protocols to ensure representativeness.

Soil Samples: Collected from the top 15–20 cm of soil using a soil auger, following a grid sampling technique.

Plant Tissue Samples: Obtained from representative plants at critical growth stages (e.g., vegetative or reproductive stages).

Laboratory Analysis:

The macronutrient levels [Nitrogen (N), Phosphorus (P), Potassium (K)] were quantified using established chemical analysis methods:

Nitrogen (N):

Method: Kjeldahl Method.

Procedure: Digestion of the sample in concentrated sulfuric acid, distillation of ammonia, and titration with a standard acid.

Phosphorus (P):

Method: Colorimetric analysis using the molybdenum blue method.

Procedure: Extraction of phosphorus with Bray or Olsen solution, followed by color development and spectrophotometric measurement.

Potassium (K):

Method: Flame photometry.

Procedure: Soil extracts were analyzed for potassium concentration using a flame photometer.

### 5.2 Exploratory crop yield data analysis

**Figure 4:**
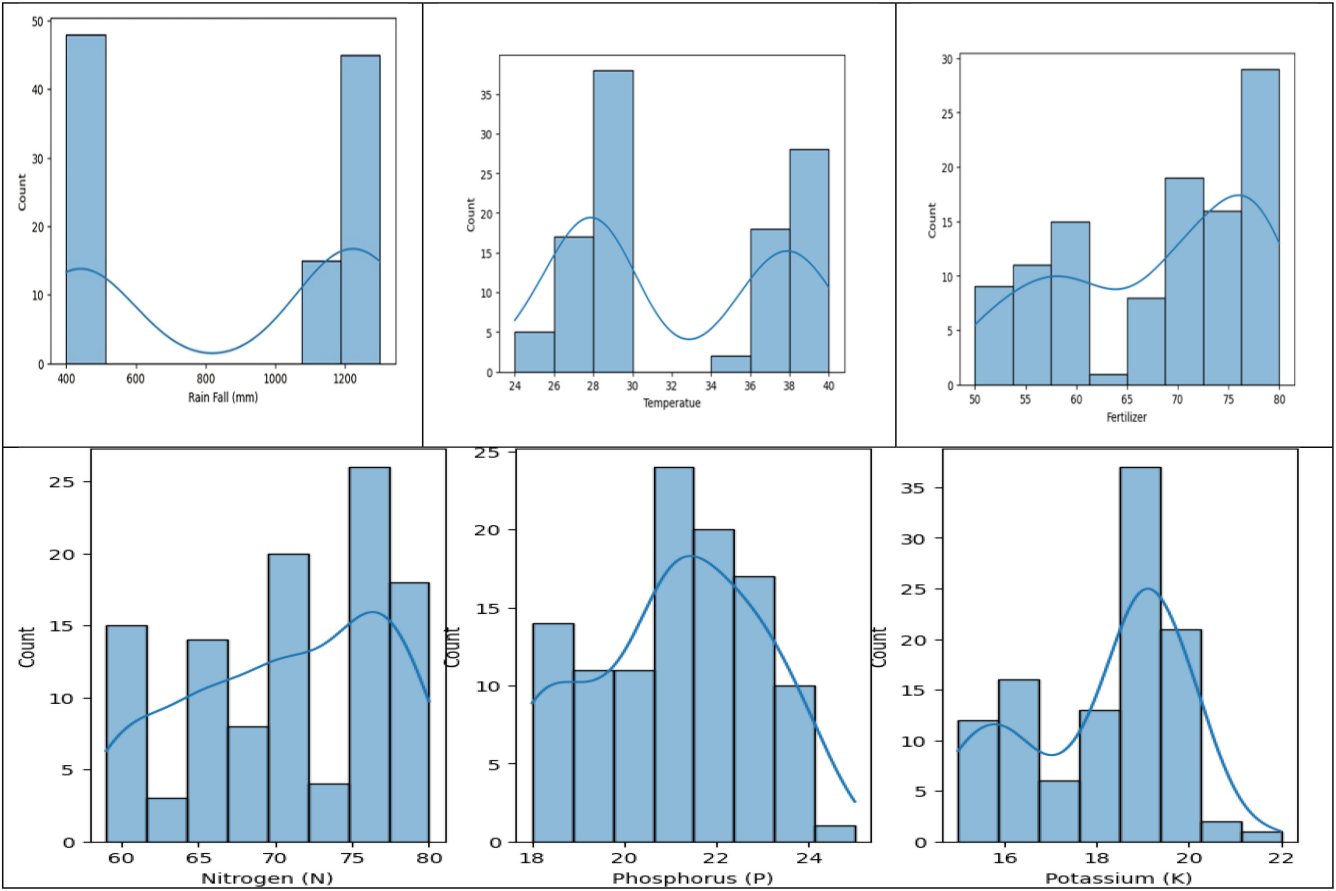
Histograms to analyze Rainfall, Temperature, and Macronutrient distribution in rice fields during two seasons (Kharif and rabi) in the region.

The dataset shows a distribution of rainfall in millimeters, with rainfall over 1100 mm and between 400 and 500 mm, suggesting different crop requirements. Fertilizer usage is divided into two categories, with the correlation between crop output and fertilizer being proportionate. The dataset is relevant to critical crop growth stages like germination, vegetative growth, and grain filling. The temperature graph shows two peaks, indicating the dataset was collected for two different crops. The macronutrient distribution shows higher nitrogen usage, less phosphorus usage, and less potassium usage, suggesting different crop requirements (**Figure 4**).

A crop yield distribution graph is shown; two smaller maxima suggest that various crops grown on the same type of soil may generate different yields.

**Figure 5:**
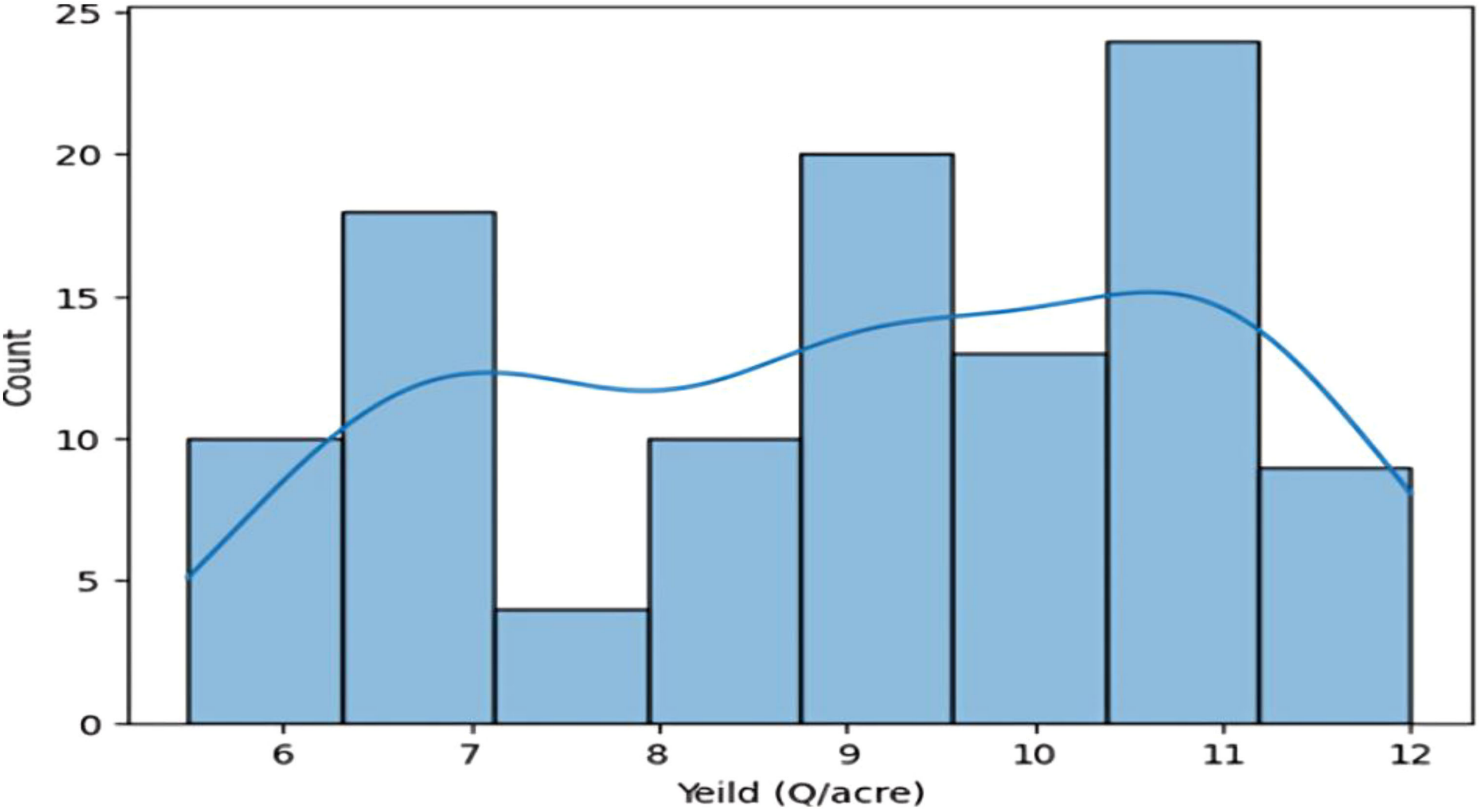
Histogram depicting relationship between crop yield and other columns (rainfall, fertiliser, temperature) during two seasons.

**Figure 6:**
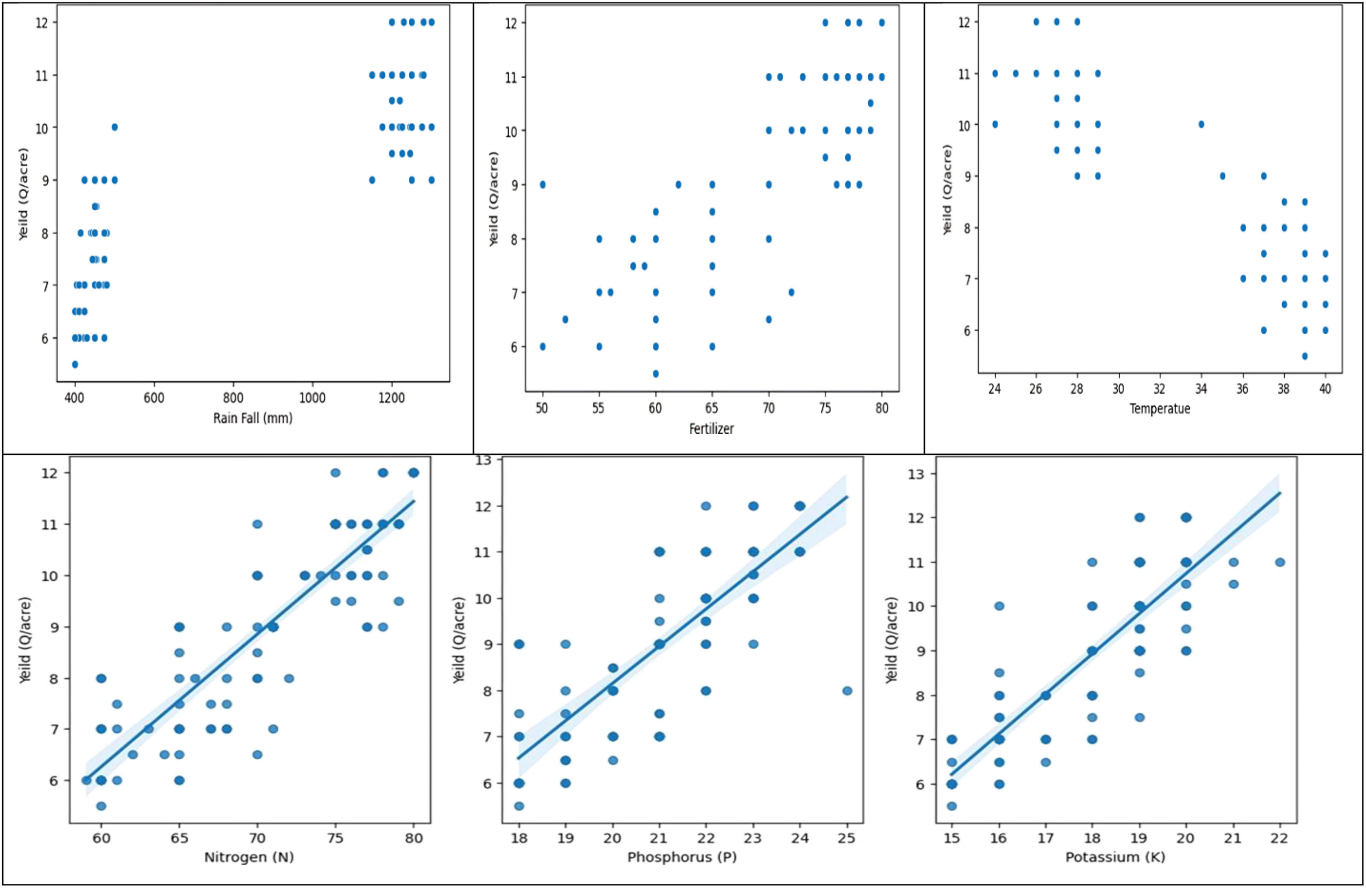
Cluster analysis of rainfall, fertilizer applied, temperature, and crop output and nutrients to understand the proportionate relationship between these factors.

The data analysis reveals two distinct crops and a relationship between crop yield and other columns. The first crop requires less rainfall, while the second requires more. Variations in crop production can be attributed to factors like soil type, temperature, fertilizer, and macronutrients. There is no direct correlation between fertilizer amount and crop output, suggesting high yields may be due to soil type and macronutrients. The first crop, rabi, is in the first cluster, while the second, kharif, is in the second. A linear relationship exists between crop output and nutrients (**Figures 5**, **6**).

**Figure 7:**
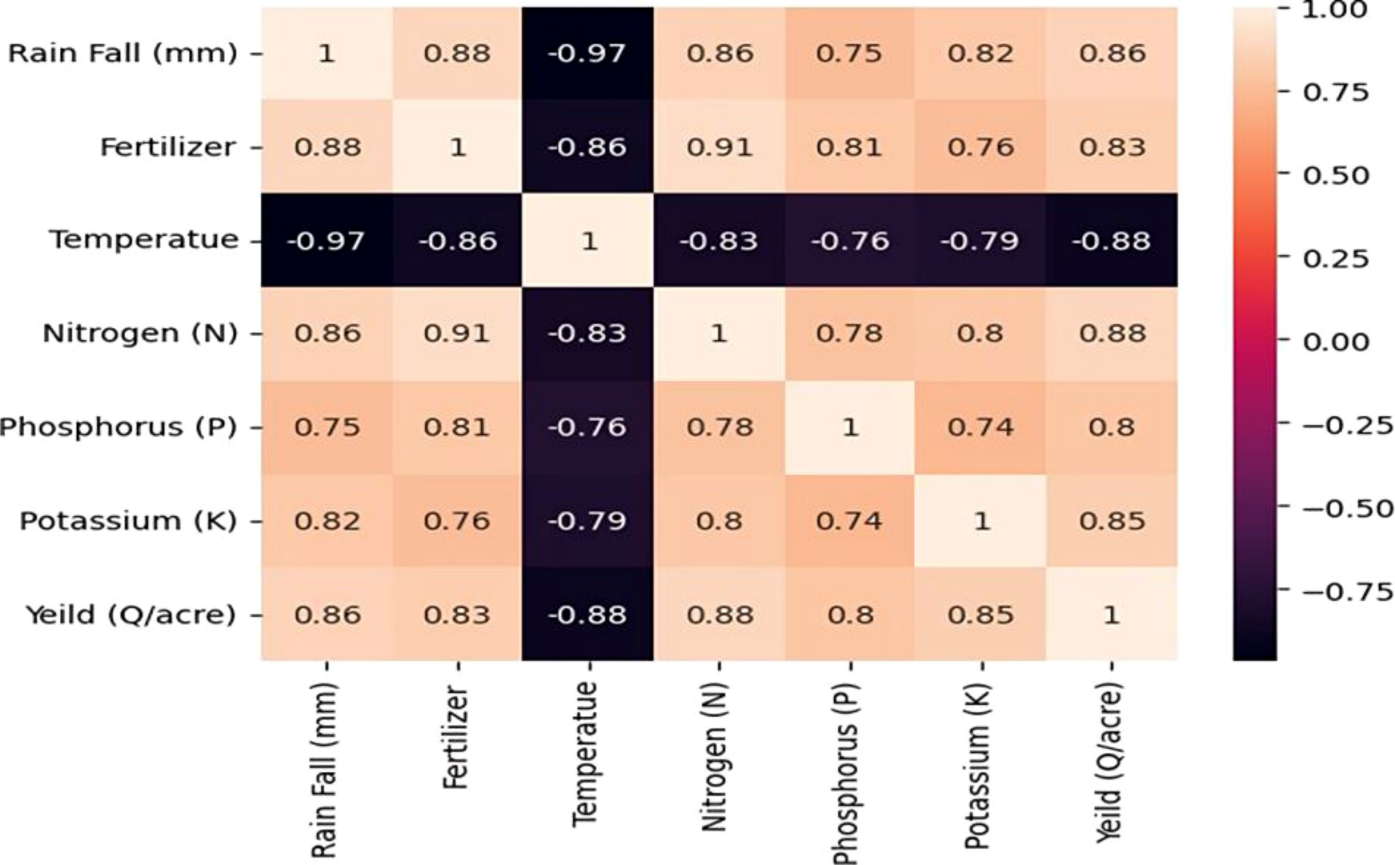
Correlation matrix heat map of crop yield prediction.

In the Correlation Matrix Heat Map, the Explanatory Data Analysis shows that the dataset was collected for two different crops. There are two clusters in the dataset for temperature, precipitation, and crop production. Nutrient levels and crop yield appear to be proportionately correlated. Nevertheless, there isn’t a straight proportionality between the crop production and the other columns. Other elements including crop breed, weather, and soil type could be to blame for this. Overall, there isn’t enough complexity in the dataset to draw firm conclusions solely from the graphs (**Figure 7**).

### 5.3 Model building

The crop yield forecast will be conducted using two models: Decision Tree Regressor and Random Forest Regressor.

**Decision Tree Regressor**: The Decision Tree Regressor is a statistical tool used to analyze the relationship between variables.

**The Decision Tree Predicted Value is 0.9279431916668135.**

**Random Forest Regressor**: The Random Forest Regressor is a statistical model used to analyze the distribution of data.

**The Random Forest Regressor Predicted Value is 0.9355014219068669.**

### 5.4 Model evaluation

**Figure 8:**
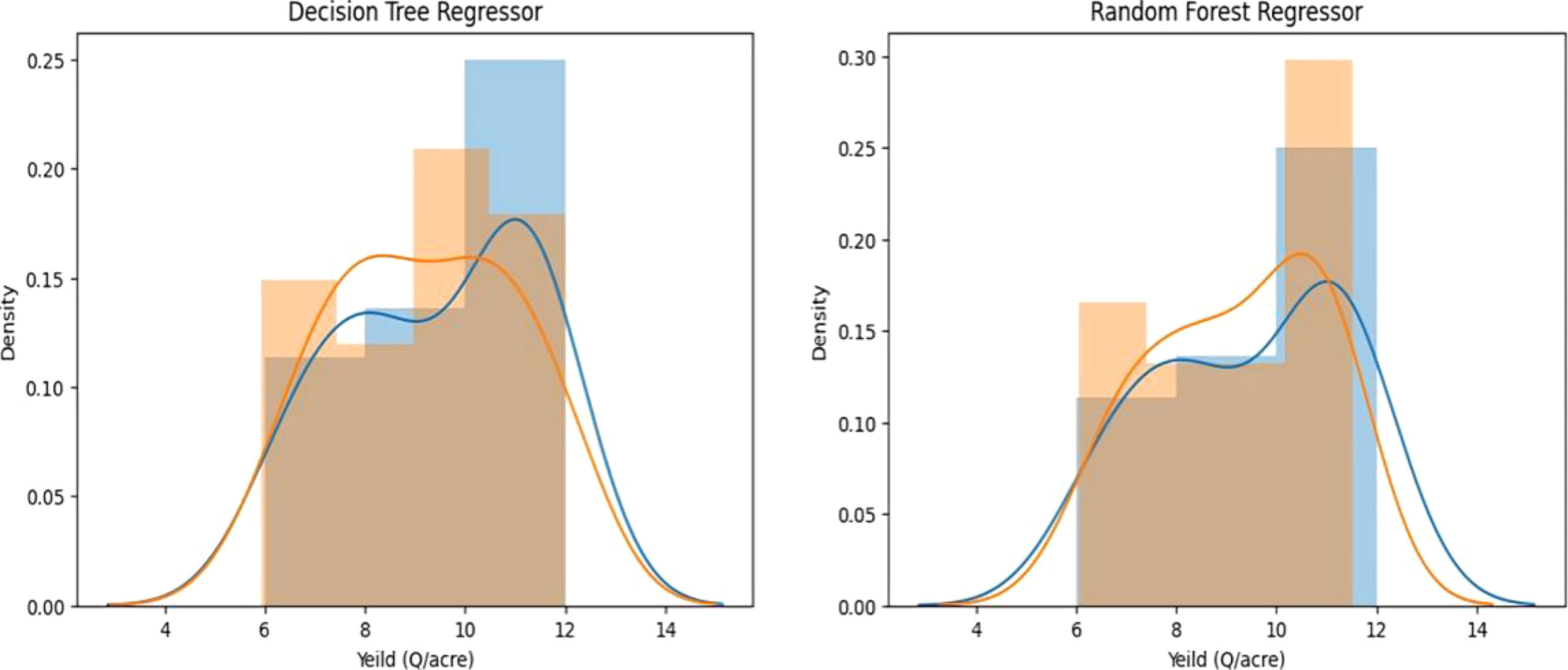
Distribution plot. Left: Decision Tree regressor; Right: Random Forest Regressor.

The text focuses on the process of model evaluation shown in **Figure 8**.

### 5.5 Evaluation metrics

Evaluation metrics are quantitative measures used to evaluate the performance and effectiveness of a Decision Regressor and Random Forest Regressor machine learning model, providing insights for comparison and improvement.

**Decision Tree Regressor:** The decision tree model is a tree-like regression tool used to predict continuous-valued outputs instead of discrete ones, consisting of leaves, branches, and nodes. This importance of rigorous evaluation of machine learning models, including metrics like R-squared, MSE, MAE to ensure their accuracy and reliability.

**Mean Squared Error:0.8250555099243105 Mean Absolute Error:0.6832293523469994**

**R2 Score:0.7709296619513172**

**Random Forest Regressor:** A random forest regressor is a kind of meta estimator that trains multiple decision tree regressors to different dataset sub-samples and uses aggregating to increase predictive accuracy and reduce over-fitting.

**Mean Squared Error:0.7108302219107439 Mean Absolute Error:0.6836246853366845 R2 Score:0.8026434375994264**

**Features of Significant Importance:** The Decision Regressor and Random Forest Regressor are crucial tools for predicting crop yield based on factors such as temperature, potassium, rain fall, nitrogen, and fertilizer.

**Figure 9:**
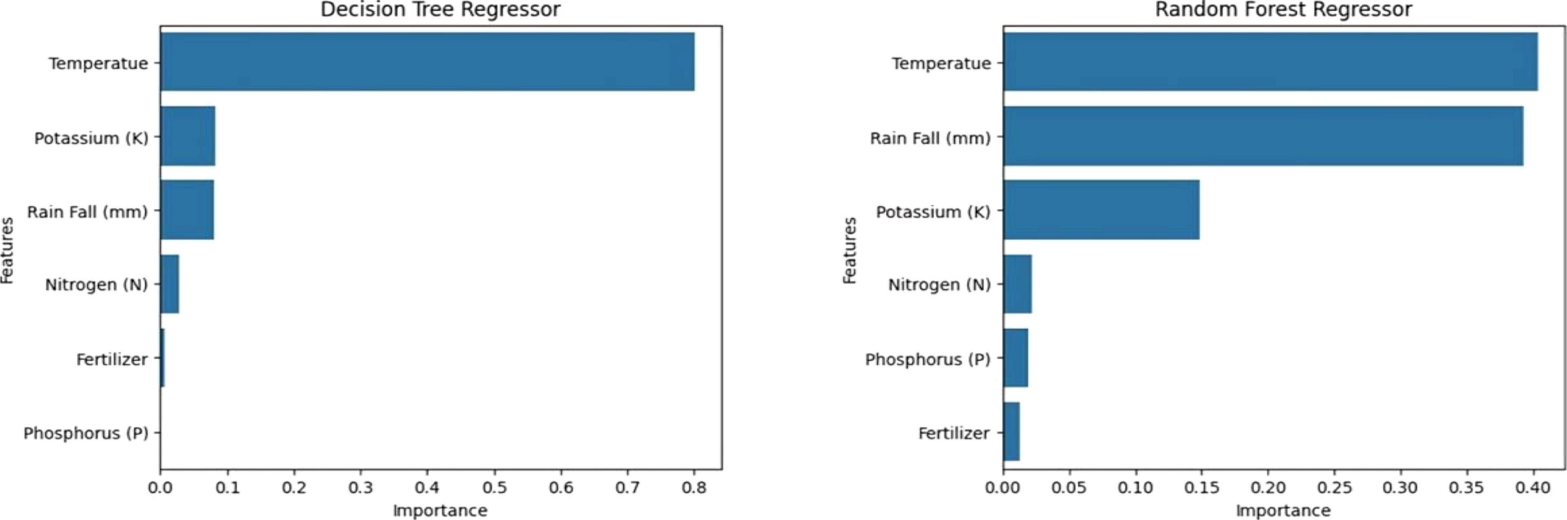
The decision regressor and random forest regressor tools for predicting crop yield (Temperature, macronutrients and rainfall).

The Exploratory Data Analysis confirms various crops with clusters in rainfall, temperature, and crop yield graphs. The dataset shows a proportional relationship between nutrients and crop yield, but not directly due to factors like soil type, weather conditions, and crop breed. The Random Forest Regressor outperformed the Decision Tree Regressor in predicting crop yield using machine learning models. The Random Forest Regressor has an R2 score of 0.802, while the Decision Tree Regressor has an R2 score of 0.77. The feature importance graph reveals that temperature holds the highest significance in predicting crop yield. The relationship between temperature and rainfall is crucial for crop yield prediction, but macro nutrients hold less significance (**Figure 9**).

**Experiment Result of LightGBM Regressor:**

**Figure 10:**
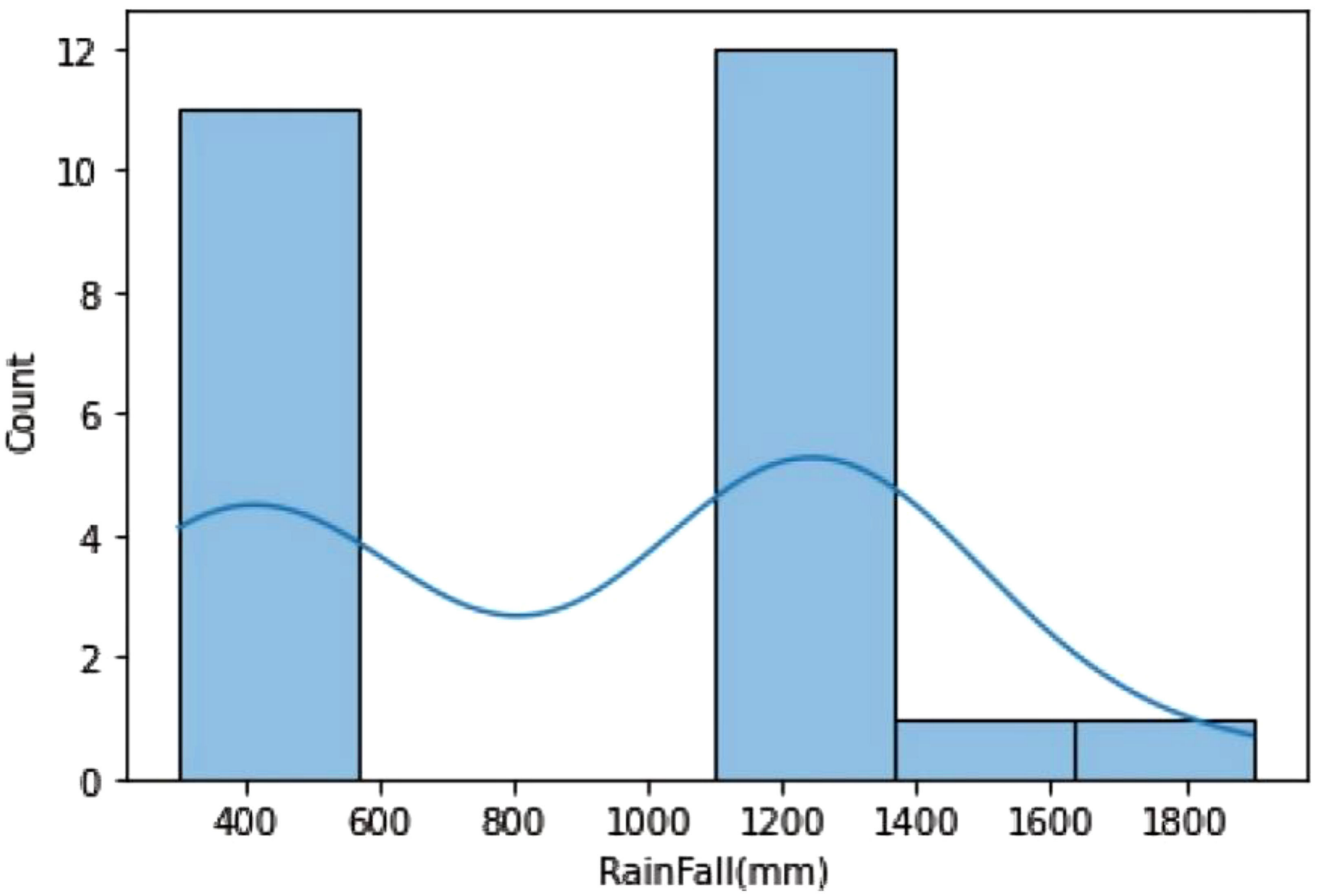
The histogram depicts irregular rainfall distribution (LightGBM Regressor).

**Rainfall Distribution:** The histogram displays irregular rainfall distribution, with over 1600 mm or 400-1600 mm falls, suggesting different crop requirements may have influenced the data collection (**Figure 10**).

**Figure 11:**
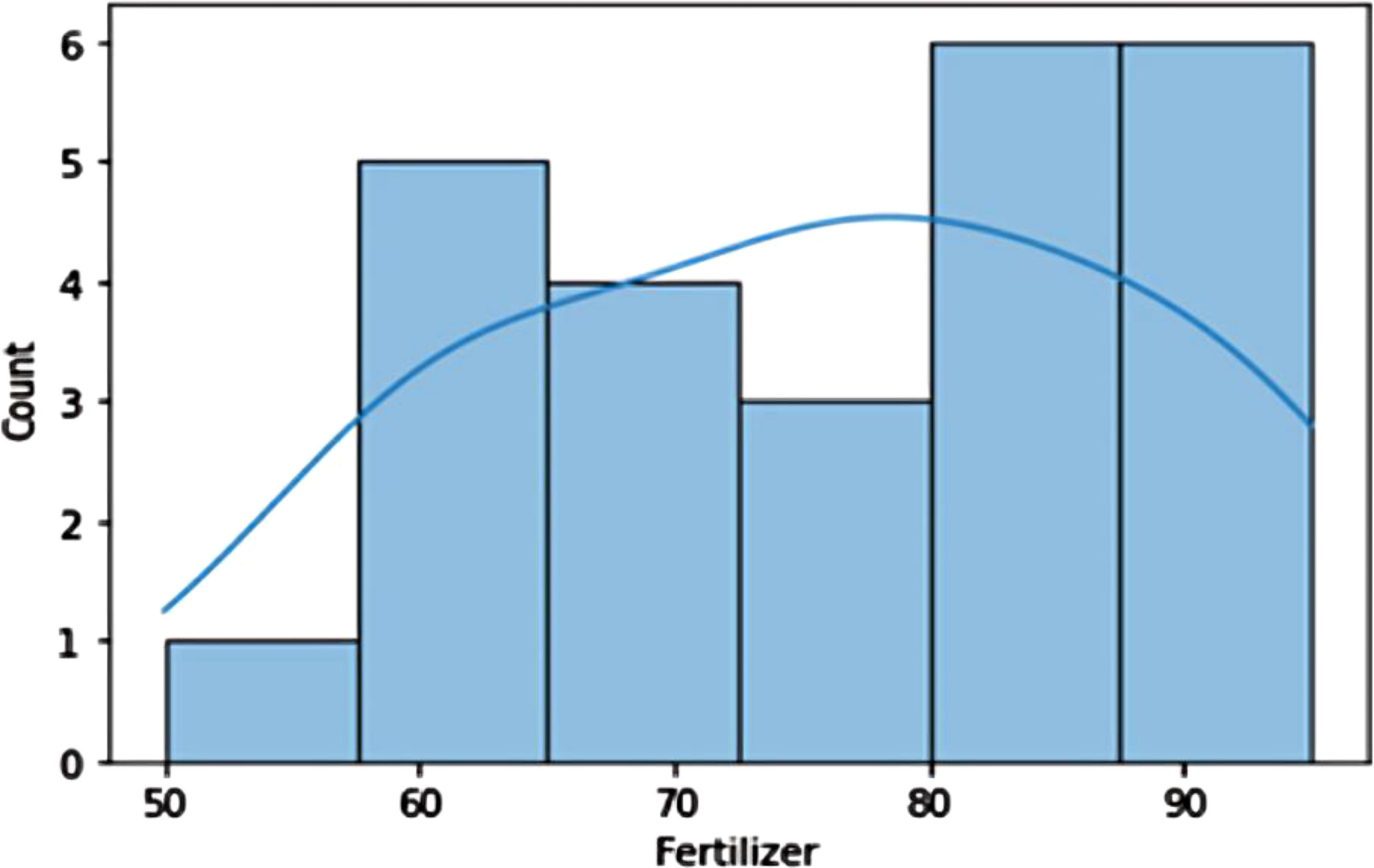
The histogram depicts the relation between fertilizer and yield proportionate.

**Fertilizer Distribution:** Fertilizer usage divided into two categories: above 65 and under 65. Crop yield is significantly impacted by fertilizer, with a potential proportionate correlation. EDA verification needed (**Figure 11**).

**Figure 12:**
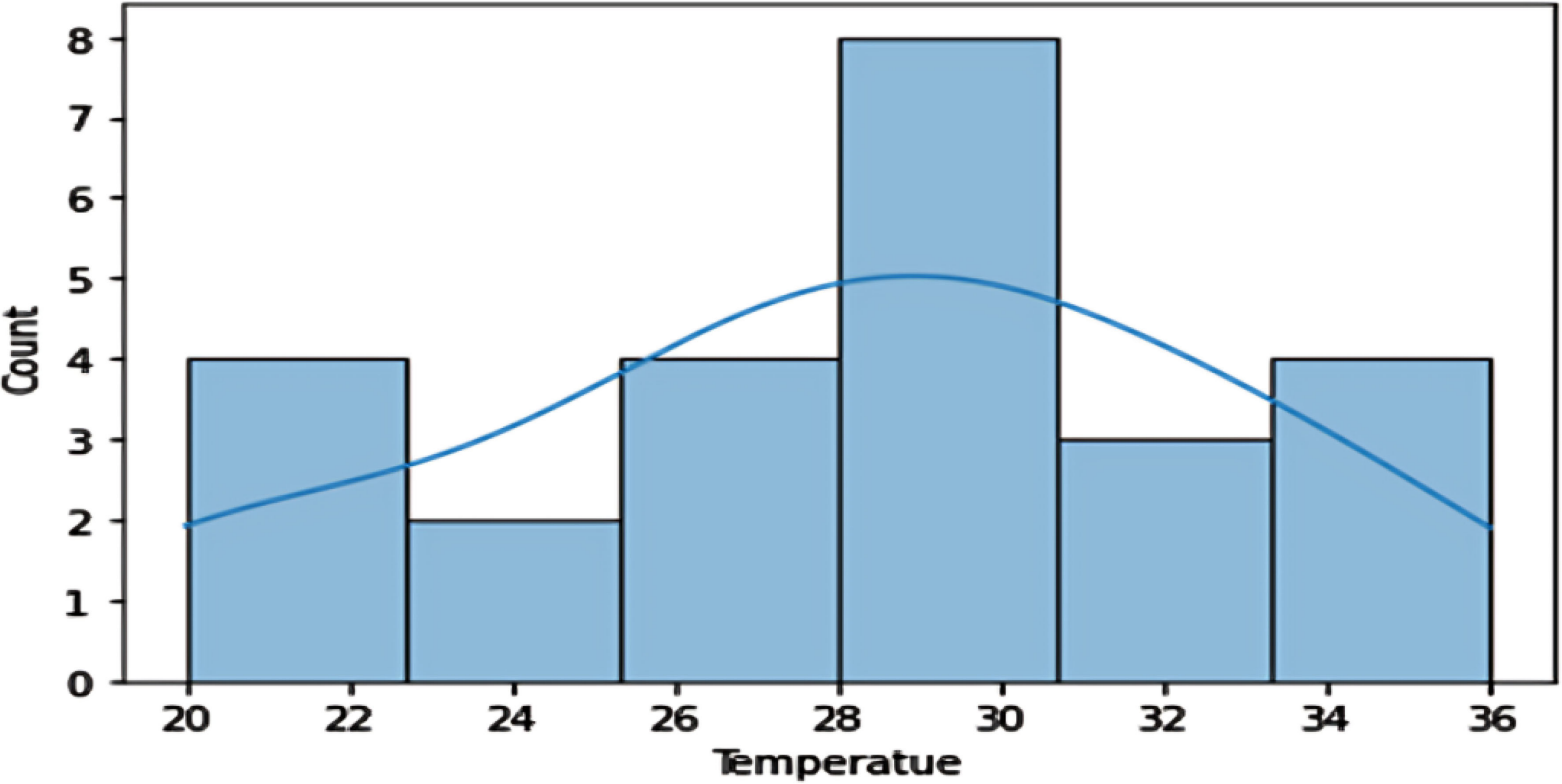
The histogram depicts the relation between Temperature and yield proportionate.

Temperature **Distribution:** The temperature graph reveals two similar peaks, suggesting the dataset may be for two different crops, possibly rabi and kharif, with an unusual temperature distribution (**Figure 12**).

**Figure 13:**
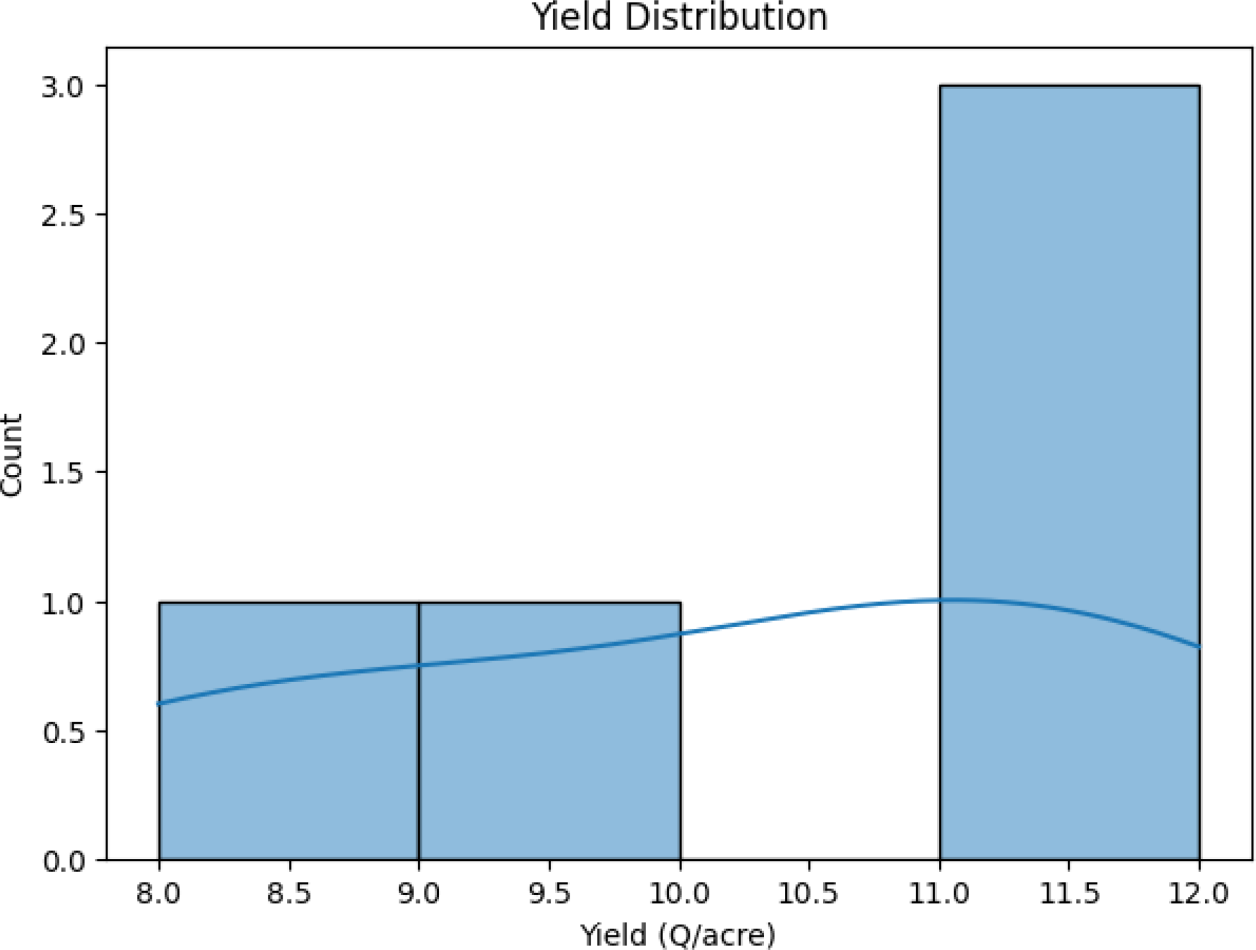
Distribution analysis between yield across different soil types.

**Yield Distribution:** The data distribution analysis reveals two distinct crops and a relationship between crop yield and other columns, suggesting varying yields across different soil types (**Figure 13**).

**Figure 14:**
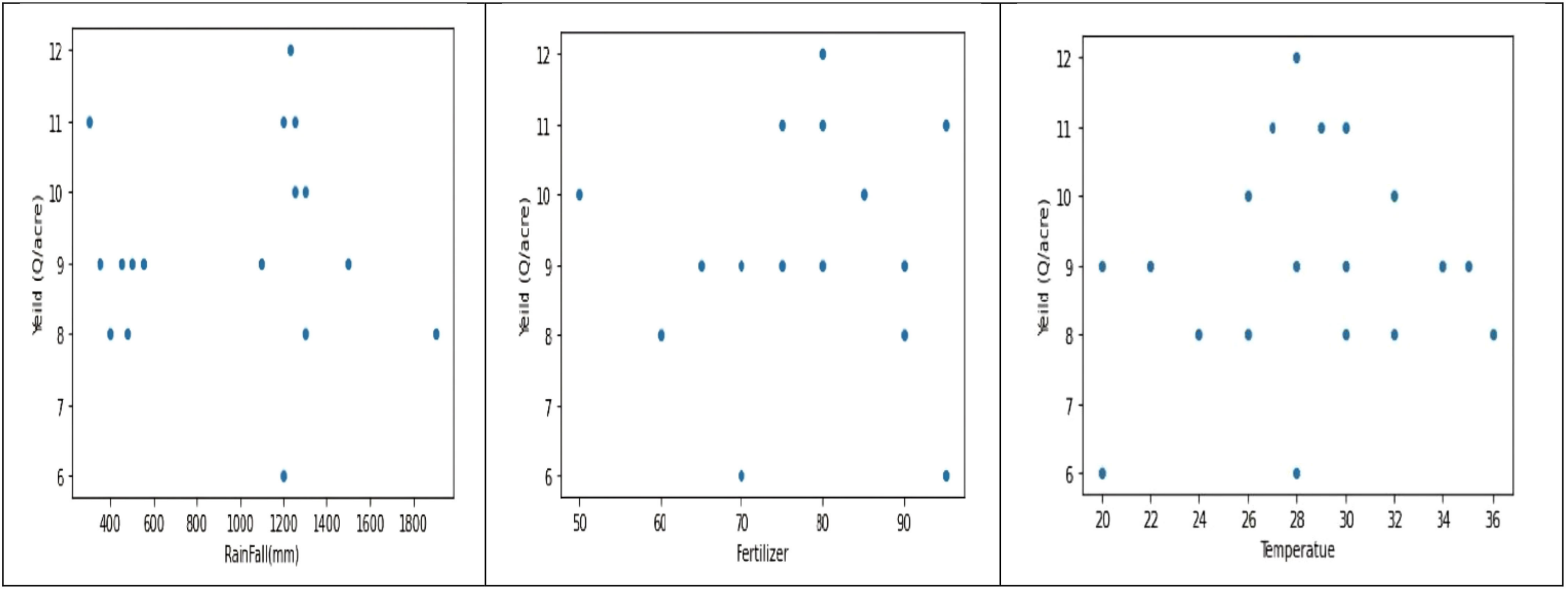
Analysis of Distribution of rainfall, fertilizers, and temperature parameters in relation to yield parameters.

**Macronutrients (NPK) Distribution:** The three graphs display the distribution of macronutrients in a crop, with nitrogen being used more frequently, phosphorus less frequently, and potassium being used less frequently, suggesting different crop requirements for nitrogen and phosphorus (**Figure 14**).

**Figure 15:**
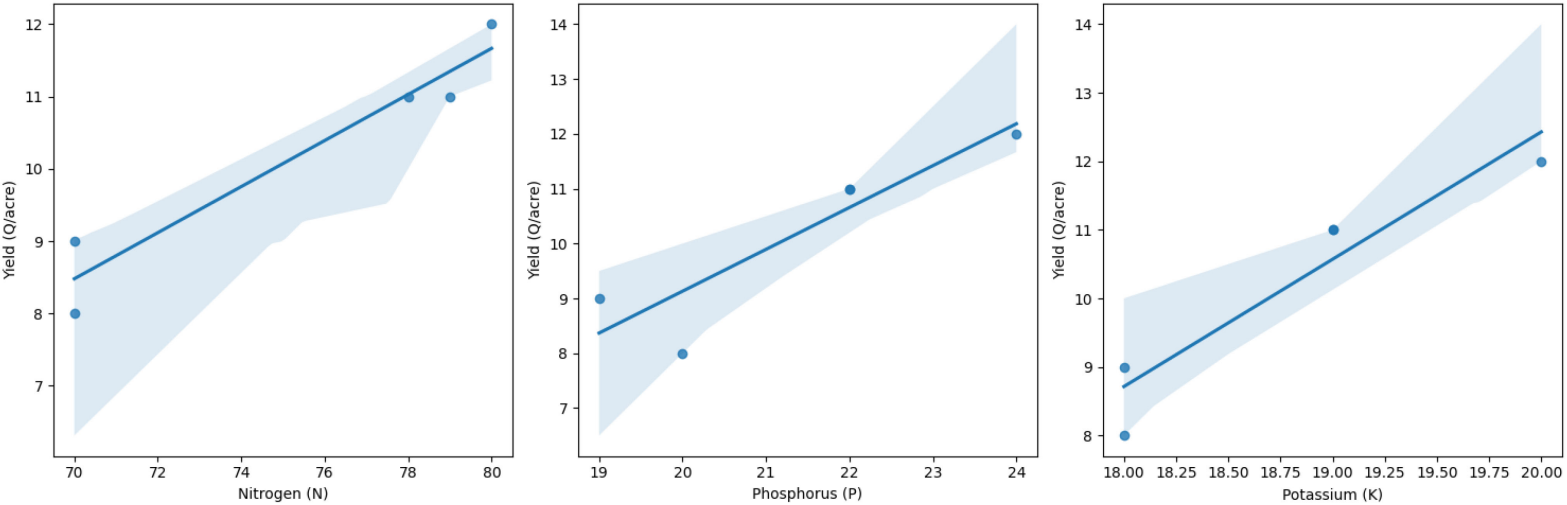
Correlation Matrix among the fertiliser application and yield parameters.

**Figure 16:**
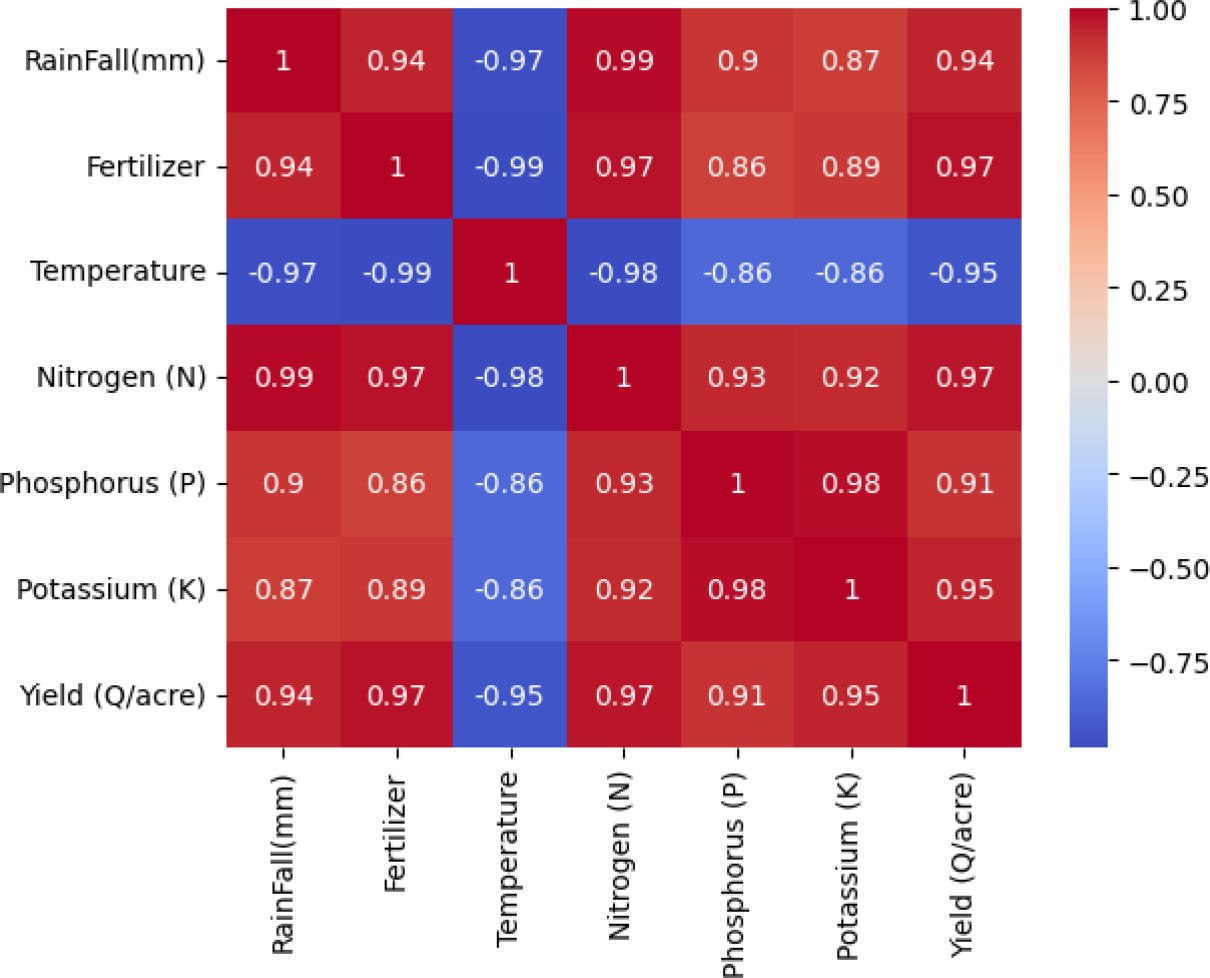
Heat Map generated between temperature, precipitation, and crop production.

**Correlation Matrix Heatmap:** The dataset, collected for two crops, shows a correlation between temperature, precipitation, and crop production. However, there’s no direct relationship between production and other columns, potentially due to factors like crop variety weather, and soil type (**Figures 15**, **16**).

**LightGBM Regressor:** LightGBM is a decision tree-based gradient boosting ensemble method used in the Train for classification and regression, optimized for high performance in distributed systems. LightGBM is a robust and efficient predictive modelling approach that starts with data preparation and feature engineering, followed by training a regressor model with specific hyperparameters and evaluation metrics.

**Mean Squared Error:2.857142857142857**

**Mean Absolute Error:1.4285714285714286**

**R2 Score:0.02941176470588225**

**RMSE:1.6903085094570331**

The LightGBM Regressor is a crucial tool for predicting crop yield based on various factors like temperature, potassium, rain fall, nitrogen, and fertilizer. The yield, temperature, and rainfall graphs’ crop clusters are validated by the exploratory data analysis. Although they are not directly caused by variables like soil type, weather, or breed, nutrients and yield are proportionate. In terms of forecasting crop yield, the LightGBM outperforms the Random Forest and Decision Tree regression models.

### 5.6 XAI techniques: implementation and interpretability

In our study, we implemented SHAP (SHapley Additive exPlanations) and LIME (Local Interpretable Model-agnostic Explanations) as XAI methods to provide clear, interpretable insights into the AI model’s crop yield predictions. These techniques allow users to understand the influence of various input features on the model’s predictions, making it possible to trust and act on these insights in real-world agricultural applications.

### 5.7 SHAP (SHapley Additive exPlanations)

SHAP assigns each feature a “Shapley value,” derived from cooperative game theory, to quantify its contribution to a particular prediction. We implemented SHAP for our LightGBM model to show how individual environmental and agronomic factors impact crop yield predictions. By visualizing Shapley values, stakeholders can see which feature such as soil moisture, temperature, or nutrient levels most significantly influence the model’s output. This helps in identifying key drivers of yield variability, aiding in decision-making, such as adjusting irrigation or fertilization practices based on the model’s predictions.

### 5.8 LIME (Local Interpretable Model-agnostic Explanations)

LIME provides local interpretability by approximating the model’s behavior in the vicinity of each prediction using a simpler, interpretable model. In our study, we used LIME to interpret individual predictions across different models. This technique was particularly valuable for identifying the conditions under which the model’s predictions might shift, offering insight into how specific environmental changes (e.g., sudden temperature rise) could influence yield estimates.

Together, SHAP and LIME improve the transparency and actionable nature of our AI predictions by offering a clear breakdown of input contributions, which is crucial in agricultural applications where practical, data-driven decisions can significantly enhance crop management and productivity. These XAI techniques ensure that our models not only predict accurately but also provide interpretable insights to support adaptive farming strategies.

## 6 Discussion

Recent studies have highlighted the potential of AI in improving crop yield predictions. For instance, deep learning models have been employed to analyze complex relationships between climatic factors and crop growth, achieving higher accuracy compared to traditional statistical methods (Raza, 2020). Furthermore, AI-driven approaches can dynamically update predictions based on real-time data, providing ongoing insights throughout the growing season.

While AI models offer robust predictive capabilities, their ‘black-box’ nature often poses challenges in interpretability. This is where Explainable AI (XAI) becomes indispensable. XAI techniques aim to elucidate the decision-making processes of AI models, providing transparency and trustworthiness. By understanding the factors that influence model predictions, stakeholders can gain confidence in the results and apply them more effectively in agricultural practices.

In this study, XAI methods such as SHAP (SHapley Additive exPlanations) and LIME (Local Interpretable Model-agnostic Explanations) were integrated to interpret the AI models’ outputs. These techniques identify key variables influencing crop yields, such as temperature, precipitation, and soil moisture levels, and explain their contributions in a human-understandable manner (Ribeiro, 2016; Lundberg and Lee, 2017). This transparency not only aids in validating model predictions but also provides actionable insights for optimizing crop management strategies.

A low R² score for LightGBM can sometimes indicate superior performance, particularly in scenarios where the dataset is noisy or contains high variance. R² measures the proportion of variance explained by the model, but it does not always capture practical utility or model robustness. LightGBM excels in handling noisy data and prioritizing meaningful patterns over fitting noise, which can result in a slightly lower R² while achieving lower Mean Squared Error (MSE) or Mean Absolute Error (MAE). This indicates that the model makes more accurate point-wise predictions. Furthermore, LightGBM’s focus on feature importance and interpretability, through tools like SHAP values, provides actionable insights that may outweigh the sole pursuit of maximizing R². In cases where generalization, feature importance, or predictions for critical scenarios are the priority, LightGBM performance may be deemed superior despite a lower R² score. This demonstrates that R² alone is not always the best metric to evaluate a model, especially in complex, real-world applications like agriculture (Hama, 2019; Nihal, 2021).

While our selected models (LightGBM, Random Forest, and Decision Tree Regressors) offer strong performance and interpretability, they do present certain limitations in complex agricultural scenarios. For instance, although these models provide insight into variable importance, they may struggle to fully capture non-linear interactions in highly complex environmental conditions, where deep learning models may excel due to their ability to learn intricate patterns from large datasets. Additionally, model accuracy can be impacted by changes in environmental variables such as unexpected weather fluctuations, soil nutrient levels, and irrigation patterns that were not captured in our dataset. These factors can influence crop yield unpredictably, presenting a challenge to models that rely on historical data patterns.

To address these constraints, future research could explore hybrid modeling approaches that combine the interpretability of tree-based models with the pattern-recognition capabilities of deep learning techniques. For instance, implementing hybrid frameworks that integrate LightGBM with neural networks may enable better handling of non-linear relationships while preserving some degree of interpretability. Additionally, advancements in data integration techniques, such as the incorporation of satellite imagery, soil data, and real-time weather monitoring, could provide our models with a more holistic understanding of crop growth environments, improving their robustness and adaptability to fluctuating conditions.

By exploring these approaches, future studies can enhance the reliability and applicability of crop yield prediction models, supporting more resilient and adaptive agricultural practices. This analysis aligns with our commitment to developing AI-driven tools that are not only accurate but also practical and interpretable for use in the agricultural domain.

The findings of this study hold several practical implications:

1. For Farmers:

Optimized Crop Management: Farmers can utilize the model’s outputs to adjust sowing times and irrigation schedules based on predicted rainfall and temperature trends.

Input Allocation: Regions with predicted nutrient deficiencies can plan targeted fertilizer applications to optimize yields while minimizing waste.

2. For Agronomists:

Site-Specific Interventions: Agronomists can leverage feature importance analysis to prioritize critical factors like soil pH or temperature while advising on crop selection and land preparation.

Pest and Disease Management: Predictive insights can assist in preemptive measures for pest outbreaks tied to climatic conditions.

3. For Policymakers:

Resource Allocation: The model enables policymakers to plan subsidies and resources for high-risk areas based on yield predictions.

Policy Formulation: Insights into environmental factors affecting yield can guide sustainable agricultural policies and investments in research for resilient crop varieties.

## 7 Conclusion

The application of AI and XAI in predicting crop yields under climate change represents a significant advancement in agricultural technology. By combining the predictive power of AI with the transparency of XAI, this approach offers a reliable and interpretable solution for addressing the challenges posed by climate variability. As these technologies continue to evolve, they hold the potential to revolutionize agricultural practices, ensuring sustainable and resilient food production systems for the future.

The integration of AI and XAI in predicting crop yields has significant implications for agricultural adaptation to climate change. By accurately forecasting yields, farmers can optimize planting schedules, select suitable crop varieties, and implement effective irrigation practices to mitigate the adverse effects of climate variability. Additionally, policymakers can use these predictions to devise strategic plans for food security, resource distribution, and disaster preparedness. Moreover, the explainability provided by XAI ensures that these predictions are not just accurate but also actionable. Farmers and agricultural advisors can understand the rationale behind the predictions, enabling them to make informed decisions that align with local conditions and sustainability goals. This approach fosters a data-driven agricultural ecosystem where decisions are backed by reliable and transparent AI insights.

The article suggests that LightGBM Regressor, Decision Tree Regressor, and Random Forest Regressor are essential tools for predicting crop yield based on various factors such as temperature, potassium levels, rainfall, nitrogen content, and fertilizer application. Exploratory Data Analysis conducted in the study confirms the existence of distinct crop clusters in rainfall, temperature, and yield graphs, revealing a consistent relationship between nutrient levels and crop yield. Interestingly, this relationship appears unaffected by soil type, weather conditions, or crop variety. The study further demonstrates the superior performance of LightGBM Regressor, Random Forest Regressor, and Decision Tree Regressor in predicting crop yield using AI and XAI models.

The research article highlights the significance of the LGBM model in machine learning contests, attributed to its enhanced accuracy, faster training time, lower memory consumption, improved overfitting control, support for parallel learning, and compatibility with datasets of varying sizes in the domain of Crop Yield Prediction in Agriculture. Additionally, the study graphically illustrates that temperature emerges as the primary factor influencing crop yield prediction. While the relationship between temperature and rainfall is deemed crucial, the contribution of macro nutrients is comparatively less significant in this context.

Machine learning is a self-motivated tool for predicting crop yields and selecting yields. It helps in intelligent decision-making in complex farming systems. The primary goal is to develop an XAI model for generating predictions of crop yield in agriculture. The Exploratory Data Analysis confirms crop clusters in rainfall, temperature, and yield graphs. Nutrients and yield are proportional, but not directly due to factors like soil type, weather conditions, or breed. The Light GBM Regressor, Random Forest Regressor outperforms Decision Tree Regressor in predicting crop yield.

## 8 Challenges and future directions

Despite the promising outcomes, several challenges remain in the application of AI and XAI in agriculture. One significant challenge is the availability and quality of data, as accurate predictions depend on comprehensive and high-quality datasets. Additionally, the computational complexity of advanced AI models requires substantial resources, which may be a barrier for widespread adoption, particularly in resource-limited regions.

Future research should focus on developing more efficient AI models that can operate with limited data and computational resources. Additionally, integrating domain knowledge from agronomy and environmental science can enhance model accuracy and relevance. There is also a need for ongoing collaboration between AI researchers, agricultural experts, and policymakers to ensure that AI technologies are tailored to meet the specific needs of different agricultural contexts.

The uniqueness of this study lies in its integration of Explainable Artificial Intelligence (XAI) with advanced machine learning (ML) methods to address the complex challenges of predicting crop yields under climate change scenarios. Unlike conventional studies that focus solely on comparing ML methods, this research emphasizes interpretability by leveraging SHAP and LIME to provide actionable insights into the interactions between key climatic variables (e.g., temperature, rainfall) and agronomic factors (e.g., soil macronutrients). The study also introduces a novel framework for using Exploratory Data Analysis (EDA) to identify critical patterns and interactions in high-dimensional agricultural data. By focusing on transparency and decision-making utility, this work transcends mere accuracy comparisons, offering a robust and scalable tool for precision agriculture, enabling policymakers and farmers to make informed, climate-resilient strategies. Additionally, the results underscore the significance of incorporating XAI into agricultural models to bridge the gap between complex AI predictions and practical implementation.

### 8.1 Enhancing model reliability and contextual relevance

While the study demonstrates significant advancements in crop yield prediction using AI and XAI, several limitations must be acknowledged. First, the models rely heavily on historical data, which may not fully capture future climate variability and extreme events. Second, while SHAP and LIME enhance interpretability, their explanations are influenced by the underlying data distribution, potentially limiting their generalizability. Additionally, the study does not incorporate socio-economic factors such as market dynamics, farmer practices, or resource availability, which are crucial for real-world applicability. Future research should focus on integrating dynamic climate models and real-time data streams to improve predictive accuracy under rapidly changing conditions. Moreover, incorporating domain-specific agronomic knowledge, such as crop phenology, pest and disease dynamics, and localized soil management practices, can enhance model reliability and contextual relevance. Interdisciplinary collaborations between data scientists and agronomists can further refine AI frameworks, ensuring that predictions align with practical farming needs. Lastly, extending the application of AI models to include prescriptive analytics providing actionable recommendations alongside predictions would significantly enhance their utility for farmers and policymakers.

## Data Availability

The original contributions presented in the study are included in the article/supplementary material. Further inquiries can be directed to the corresponding author.

## Footnotes

1 Explainable Artificial Intelligence (XAI): XAI refers to methods and techniques in artificial intelligence that allow models to be more interpretable, providing human-understandable explanations for their predictions. This transparency is crucial for trust and informed decision-making in fields like agriculture. SHAP (SHapley Additive exPlanations): SHAP is an XAI technique based on game theory that quantifies the contribution of each input feature to the output of a predictive model. It provides a comprehensive view of how features influence the model's predictions. LIME (Local Interpretable Model-agnostic Explanations): LIME is an XAI approach that creates interpretable approximations of machine learning model behavior for individual predictions by building local surrogate models.

## References

[B1] Bezner Kerr R. Hasegawa T. Lasco R. Bhatt I. Deryng D. Farrell A.. (2022). “Chapter 5: Food, Fibre, and Other Ecosystem Products,” in Climate Change 2022: Impacts, Adaptation and Vulnerability. Contribution of Working Group II to the Sixth Assessment Report of the Intergovernmental Panel on Climate Change. Eds. Pörtner H.-O. Roberts D. C. Tignor M. Poloczanska E. S. Mintenbeck K. Alegría A. Craig M. Langsdorf S. Löschke S. Möller V. Okem A. Rama B. (Cambridge University Press, Cambridge, UK and New York, NY, USA). doi: 10.1017/9781009325844.007

[B2] Breiman L. (2017). Classification and Regression Trees (New York: Routledge). doi: 10.1201/9781315139470

[B3] Gohel P. (2021). Explainable AI: current status and future directions. arXiv. Available at: https://arxiv.org.

[B4] Graves A. R. Hess T. Matthews R. B. Stephens W. Middleton T. (2002). Crop simulation models as tools in computer laboratory and classroom-based education. J. Natural Resour. Life Sci. Educ. 31, 48–54. doi: 10.2134/jnrlse.2002.0048

[B5] Hama S. J. (2019). Correlation and path coefficient analysis for seed yield and yield components in chickpea under rainfed condition. J. Kerbala Agric. Sci. 6, 26–35. doi: 10.59658/jkas.v6i1.595

[B6] Jame Y. W. Cutforth H. W. (1996). Crop growth models for decision support systems. Can. J. Plant Sci. 76, 9–19. doi: 10.4141/cjps96-003

[B7] Jehad A. (2012). Random forests and decision trees, IJCSI international. J. Comput. Sci. Issues 9, 1694–0814. Available at: www.IJCSI.org (Accessed September, 2012).

[B8] Kamilaris A. Prenafeta-Boldú F. X. (2018). Deep learning in agriculture: A survey. Comput. Electron. Agric. 147, 70–90. doi: 10.1016/j.compag.2018.02.016

[B9] Khaki S. (2019). Crop yield prediction using deep neural networks. Front. Plant Sci. 10. doi: 10.3389/fpls.2019.00621 PMC6540942 31191564

[B10] Khaki S. Khalilzadeh Z. (2022). Applications of Convolutional Neural Networks in agriculture: A survey. Front. Plant Sci. 13. doi: 10.3389/fpls.2022.1023515

[B11] Kussul N. Lavreniuk M. Skakun S. Shelestov A. Y. (2017). Deep learning classification of land cover and crop types using remote sensing data. IEEE Geosci. Remote Sens. Lett. 14, 778782. doi: 10.1109/LGRS.2017.2681128

[B12] Linardatos P. Papastefanopoulos V. Kotsiantis S. (2021). Explainable AI: A review of machine learning interpretability methods. Entropy 23, 18. doi: 10.3390/e23010018 PMC7824368 33375658

[B13] Lundberg S. M. Lee S. I. (2017). A unified approach to interpreting model predictions. Adv. Neural Inf. Process. Syst. 30, 4765–4774. doi: 10.48550/arXiv.1705.07874

[B14] Nihal D. (2021). Linear Decision Tree Regressor: Decision Tree Regressor Combined with Linear Regressor (GITHUB).

[B15] Panel Saranya A. Subhashini R. (2023). A systematic review of Explainable Artificial Intelligence models and applications: Recent developments and future trends. Decision Anal. J. 7. doi: 10.1016/j.dajour.2023.100230

[B16] Purna Syam Chand S. Divya G. (2022). A Light Gradient Boosting Machine Regression Model for Prediction of Agriculture Insurance Cost over Linear Regression, Advances in Parallel Computing Algorithms, Tools and Paradigms. Ed. Hemanth D. J.. (Amsterdam, Netherlands: IOS Press). doi: 10.3233/APC220027

[B17] Rasheed A. Xiaodong L. Kashif S. (2023). Temporal convolutional networks for crop yield prediction using remote sensing data. IEEE Trans. Geosci. Remote Sens. 61, 19. doi: 10.1109/TGRS.2023.3141236

[B18] Raza M. Q. (2020). Artificial intelligence-based prediction of crop yields using climatic parameters. J. Agron. Crop Sci.

[B19] Rezaei E. E. Siebert S. Müller C. (2021). Integrated assessment of climate change impacts on crop yields and crop water productivity in Europe. Agric. Syst. 193, 103210. doi: 10.1016/j.agsy.2021.103210

[B20] Ribeiro M. T. (2016). “Why should I trust you?”: Explaining the predictions of any classifier,” in Proceedings of the 22nd ACM SIGKDD International Conference on Knowledge Discovery and Data Mining. (San Francisco, USA: Association for Computing Machinery (ACM)).

[B21] van Klompenburg T. Kassahun A. Catal C. (2020). Crop yield prediction using machine learning: A systematic literature review. Comput. Electron. Agric. 177, 105709. doi: 10.1016/j.compag.2020.105709

